# Comprehensive Management of Different Types of Pelvic Fractures Through Multiple Disciplines: A Case Series

**DOI:** 10.3390/jcm14155593

**Published:** 2025-08-07

**Authors:** Bharti Sharma, Samantha R. Kiernan, Christian Ugaz Valencia, Omolola Akinsola, Irina Ahn, Agron Zuta, George Agriantonis, Navin D. Bhatia, Kate Twelker, Munirah Hasan, Carrie Garcia, Praise Nesamony, Jasmine Dave, Juan Mestre, Zahra Shafaee, Suganda Phalakornkul, Shalini Arora, Saad Bhatti, Jennifer Whittington

**Affiliations:** 1 Department of Surgery, NYC Health & Hospitals/Elmhurst, Queens, NY 11373, USA; akinsolo@nychhc.org (O.A.); agriantg@nychhc.org (G.A.); bhatian1@nychhc.org (N.D.B.); hassanm14@nychhc.org (M.H.); carrie.garcia@nychhc.org (C.G.); nesamonp@nychhc.org (P.N.); davej@nychhc.org (J.D.); mestreju@nychhc.org (J.M.); shafaeez1@nychhc.org (Z.S.); phalakos@nychhc.org (S.P.); arorash@nychhc.org (S.A.); bhattisa@nychhc.org (S.B.); harrisj20@nychhc.org (J.W.); 2 Department of Surgery, Icahn School of Medicine at Mount Sinai Hospital, New York, NY 10029, USA; christian.ugazvalencia@mountsinai.org; 3 Department of Medicine, Touro College of Osteopathic Medicine–Harlem, New York, NY 10027, USA; skiernan@student.touro.edu; 4 Department of Medicine, St. George’s University, Grenada, Great River, NY 11739, USA; iahn@sgu.edu (I.A.); azuta@sgu.edu (A.Z.)

**Keywords:** pelvis, multiple trauma, sacrum, fractures, multiple, patient care team

## Abstract

**Background:** Pelvic fractures are complex injuries often associated with significant morbidity and mortality, requiring multidisciplinary management. This case series highlights the presentation, management strategies, and outcomes of patients with pelvic fractures treated at our institution. **Methods:** The medical records of 13 patients diagnosed with pelvic fractures from 1 January 2020 through 31 December 2023 were retrospectively reviewed. Demographic data, mechanism of injury, fracture pattern, associated injuries, treatment modalities, and outcomes were analyzed. **Results:** A total of 13 patients were included in the study, with ages ranging from 18–95 years. Six of the patients were male and seven were female. The most common mechanisms of injury were falls and pedestrians struck by vehicles. Associated injuries included traumatic brain injury (TBI), fractures including extremities, ribs, and vertebrae, visceral injury, and spinal cord injury. Treatment strategies ranged from conservative, non-surgical management to operative intervention, including interventional radiology embolization, external traction, open reduction and internal fixation (ORIF), and percutaneous screw stabilization. Additional interventions included chest tube placement, exploratory laparotomy, and craniectomy. Two patients died while in the hospital, one was discharged to a shelter, and the remaining 10 were discharged to various inpatient rehab facilities. **Conclusions:** Pelvic fractures pose significant clinical challenges due to their complexity and associated injuries. This case series underscores the importance of multidisciplinary intervention and treatment strategies in optimizing outcomes. Further studies should focus on the effectiveness of interventions, utilization of new technology, and multidisciplinary team planning.

## 1. Background Information

Pelvic fractures are among the most challenging injuries encountered in trauma care due to their complexity, potential for life-threatening complications, and significant impact on a patient’s functional outcome. They are also often associated with other traumatic injuries. Pelvic injuries account for up to 10% of trauma admissions [1,2,3] and pelvic ring fractures have an incidence of approximately 20 cases per 10,000 persons per year [1,4,5,6]. Reported mortality rates can vary greatly depending on the type and severity of injury, mechanism of injury, comorbid injuries, and baseline patient heath status, ranging anywhere from 5–66% [1,6,7,8,9,10,11,12,13], with open fractures having an overall higher mortality compared to closed fractures [10]. Hemodynamically unstable patients also have a higher mortality rate [1]. This mortality is largely attributable to hemorrhage, which can come from a multitude of arterial and venous, as well as soft tissue structures in the pelvis [1,7,8,10,14]. Additionally, multiple visceral structures within the pelvis are at risk of being damaged. Because of these intricacies, a multidisciplinary approach is often necessary to care for these patients.

The most common mechanisms of injury for pelvic fractures are falls and road traffic accidents [1,9,10,15]. Many of these fractures are associated with high-energy mechanisms, such as motor vehicle collisions, pedestrians struck by vehicles, or falls from great heights, though low-energy trauma like falls from standing can also cause pelvic fractures in certain populations [1,15]. There is no one agreed-upon system to classify pelvic fractures, though three commonly used methods include the Tile classification, the Young and Burgess classification, and the World Society of Emergency Surgery (WSES) classification. The Tile classification system divides pelvic fractures into three categories: Type A are stable, Type B have rotational instability but vertical stability, and Type C have both rotational and vertical instability [6]. The Young and Burgess classification system is based on the direction of force that caused the injury and is divided into four categories: lateral compression (LC), anteroposterior compression (APC), vertical shear (VS), and combined mechanism injury (CMI) [6]. The WSES classification system divides pelvic fractures into three classes: minor or grade I, which are mechanically and hemodynamically stable, moderate or grade II and III, which are mechanically unstable but hemodynamically stable, and severe or grade IV, which are hemodynamically unstable regardless of mechanical stability [6,14]. Additionally, there are classification systems to categorize sacral fractures specifically. One such system, the AO Spine Sacral and Pelvic Classification System, categorizes sacral fractures into three main divisions: type A or lower sacrococcygeal, which have no impact on the stability of the posterior pelvic ring or spine, type B or posterior pelvic, which disrupt the posterior pelvic complex while preserving spinopelvic stability, and type C or spinopelvic, which affect spinopelvic stability [16].

Management of pelvic fractures includes treating the fracture(s) themselves as well as treating the possible associated soft tissue and visceral injuries. Initial management largely depends on the stability of the patient on presentation. For patients who are unstable or who are suspected to have a hemorrhagic injury, evaluation with rapid imaging modalities, such as pelvis radiographs or Focused Assessment with Sonography for Trauma (FAST), is preferred [3,6,10,14], and pelvic binding is recommended [6,14]. For stable patients, CT imaging is preferred as it is more sensitive and able to visualize fractures that may be missed on plain film radiographs, and contrast may be utilized to assess vascular and visceral injury [3,6,10,14]. Further management may include a range of treatment modalities depending on individual patient needs, including non-operative observation, angioembolization, external fixation, pelvic packing, REBOA, internal fixation, and occupational and physical therapy [6,10,14].

This case series provides 13 examples of pelvic and sacral fractures with a wide variety of injury mechanisms and, by extension, a varied approach to management by multidisciplinary teams. Patient demographics, presenting complaints, detailed clinical history, physical examination findings, relevant investigations (labs, imaging), management plan, and hospital course were reviewed. By examining actual cases, we aim to highlight the nuances of diagnosis, stabilization, surgical intervention, and rehabilitation. Given the variability and complexity of pelvic fractures, sharing these cases brings valuable insight into barriers encountered by different members of the care team and how cases are managed when there are conflicting management plans. These examples contribute to ongoing clinical discussions by illustrating real-world challenges not often captured by larger studies or guidelines.

## 2. Methods

This was a single-center retrospective review conducted at a level 1 trauma center in Queens, New York City, from 1 January 2020 through 31 December 2023. Patients were identified through the National Trauma Registry of the American College of Surgeons (NTRACS) Database as those who sustained a pelvic fracture (defined as a fracture to any of the bones involved in the pelvic ring, including the sacrum, ilium, acetabulum, ischium, and pubis). Patients were excluded if they did not sustain a fracture to this region. A chart review was performed for each of these patients and information such as demographic data, initial presentation, mechanism of injury, associated injuries, imaging findings, hospital course, disposition, and outcomes was collected. Additionally, a review of radiologic images was conducted, and exemplary images were collected.

## 3. Cases Presentation

### 3.1. Case 1

#### 3.1.1. Presentation

A 52-year-old man with a past medical history of polysubstance abuse and bipolar 1 disorder presented to the emergency department after a fall from over 25 feet with unknown impact and unknown loss of consciousness. On presentation, the patient endorsed pain “everywhere,” and did not remember the fall or the events leading up to the fall. He was exhibiting signs of intoxication and was unable to supply a reliable history of his injuries. Physical examination of the head revealed raccoon eyes, contusions, and lacerations of the head. Examination of the nose revealed nasal deformity, signs of nose injury, and nasal tenderness. Pulmonary examination revealed tachypnea. Musculoskeletal examination revealed left knee, left upper leg, and left lower leg tenderness. Skin revealed signs of injury, laceration, and wounds. Neurological exam revealed confusion and a Glasgow Coma Scale score of 14. The FAST exam was negative.

#### 3.1.2. Imaging

**Figure 1:**
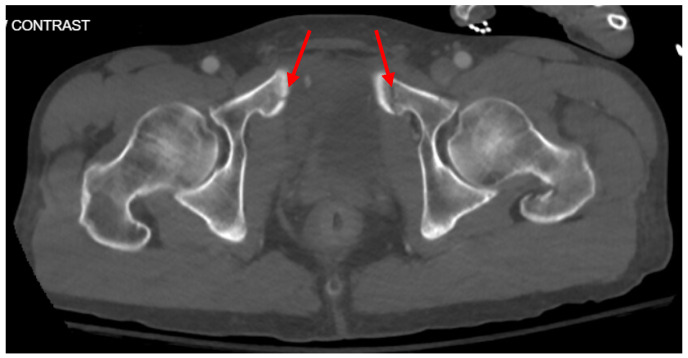
CT abdomen/pelvis images of the described fractures in case 1. Red arrows are used to indicate the location of pelvic fractures for easier identification.

Pelvic imaging revealed acute fractures involving the right superior pubic ramus and left inferior pubic ramus, an acute comminuted avulsed fracture of the left sacral wing in the superior and midportion (Figure 1). Additional injuries included multiple facial fractures, left distal radius fracture, left femur fracture, and left tibial shaft fracture.

#### 3.1.3. Management and Outcome

Given the patient’s multiple injuries, he was admitted to the SICU for close monitoring and treatment. Orthopedic surgery was consulted for management of the extremity and pelvic fractures, and recommended non-weight bearing for the left lower extremity with a plan to go to the OR the following day, though this was initially deferred due to recent cocaine use. Neurosurgery was consulted regarding the sacral fracture and recommended non-weight bearing with consideration for surgical vs non-surgical stabilization. He was eventually taken to the OR on hospital day 9 for percutaneous left SI screw placement with left L5 instrumentation. Other surgical managements included an open reduction internal fixation of the left femur and insertion of an intramedullary nail of the left tibia. His hospital course was complicated by aspiration pneumonia requiring intubation as well as continued disorientation and agitation, for which psychiatry was consulted for management. Facial fractures were recommended to be managed on an outpatient basis by plastic and ophthalmology. He was evaluated by physical medicine and rehabilitation and discharged to subacute rehab on hospital day 26.

### 3.2. Case 2

#### 3.2.1. Presentation

A 62-year-old female with a past medical history of hypertension and hyperlipidemia was brought in by EMS after being struck by a vehicle going approximately 25 mph. The accident was witnessed by her husband. She was thrown backwards and hit the back of her head, and lost consciousness for about 5 min. She was not complaining of any pain on presentation, but was not able to recall the incident. The exam revealed a GCS of 15, an airway patent and self-maintained, and she was moving all extremities spontaneously. She was noted to have a 3 cm left lateral scalp laceration and abrasions to the left ankle, anterior foot, and right knee. She had multiple episodes of non-bloody, nonbilious emesis during the exam. No other significant findings were noted. The FAST exam was negative.

#### 3.2.2. Imaging

**Figure 2:**
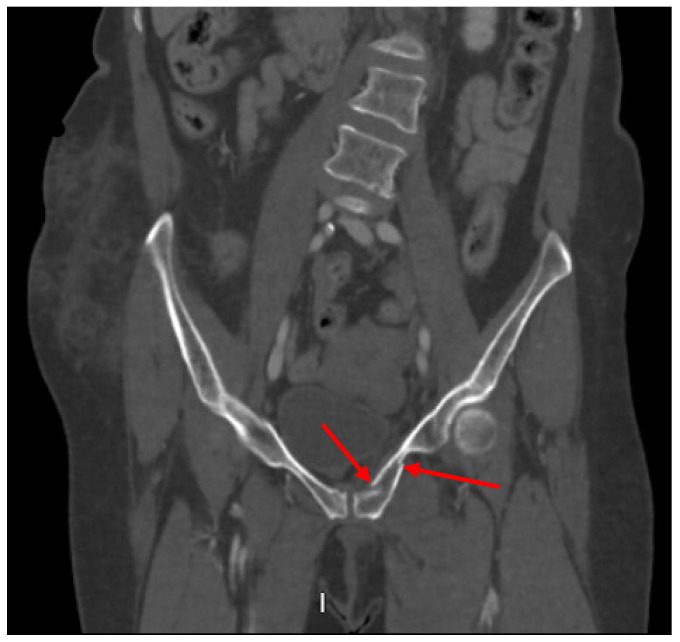
CT abdomen/pelvis images of the described fractures in case 2. Red arrows are used to indicate the location of pelvic fractures for easier identification.

Pelvic imaging showed an acute impacted fracture involving the left superior and inferior pubic rami (Figure 2). She was also found to have diffuse asymmetric areas of subarachnoid blood throughout the frontal and parietal regions, with additional blood within the third and fourth ventricles and the posterior aspect of the occipital on CT head.

#### 3.2.3. Management and Outcome

While in the ED, the patient’s mental status quickly deteriorated, and her pressure dropped to 72/50. Mass transfusion protocol was initiated, she was intubated, and she was found to have increased subarachnoid bleeding on repeat imaging, and neurosurgery was consulted. During her hospital course, the patient was also found to have a right lateral tibial plateau fracture and bilateral sacral ala fractures; orthopedic surgery recommended non-weight bearing, and neurosurgery recommended bed rest. Further management included tracheostomy on hospital day 8 and PEG on hospital day 19. The patient was seen by physical medicine and rehabilitation, who recommended physical and occupational therapy, and she was discharged to TBI rehab on hospital day 39.

### 3.3. Case 3

#### 3.3.1. Presentation

An 18-year-old female with no past medical history was brought in by EMS after being found down in the snow under a fire escape from a fall from a third-story window. She was initially somnolent and not oriented but became more responsive in the trauma bay. She was complaining of severe back pain. She reported that she jumped from her fire escape. On presentation, she was hypotensive with SBP in the 60s, so the massive transfusion protocol was initiated, and a pelvic binder was placed. Physical exam was remarkable for externally rotated right hip and abrasion of the left glute, tenderness to palpation in the thoracic and lumbar spine, and questionable pelvic stability. The patient was moving her upper extremities but not her lower extremities. FAST was unremarkable.

#### 3.3.2. Imaging

**Figure 3:**
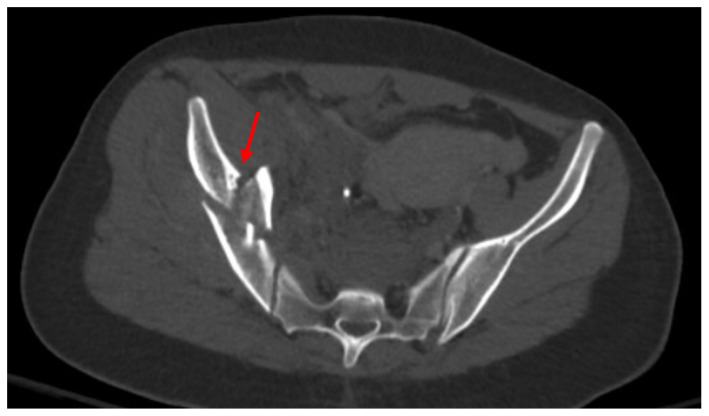
CT abdomen/pelvis images of the described fractures in case 3. Red arrows are used to indicate the location of pelvic fractures for easier identification.

Pelvic imaging showed a fracture of the distal portion of the sacrum with anterior displacement, a comminuted fracture through the right iliac bone with moderate displacement, a moderately displaced fracture through the right inferior pubic ramus, and mildly displaced fractures of the right anterior and posterior acetabulum that involve the articular surface. There is a hemorrhage surrounding the right iliac fracture, a small volume hemorrhage in the right pelvic sidewall and the presacral space, and a small volume hemorrhage surrounding the urinary bladder (Figure 3).

Extensive additional injuries were also found, including subdural hematomas without mass effect, compression fractures of T6–8, T11, and T12, L2 and L3 burst fractures with retropulsion of fracture fragments completely effacing the spinal canal, left 4th, 5th, and 6th rib fractures, nondisplaced manubrial fracture, and left-sided pneumothorax.

#### 3.3.3. Management and Outcome

A right femoral traction pin was placed by an orthopedic surgeon in the ED. Neurosurgery was consulted for the patient’s multiple spinal fractures and brought the patient to the OR for L1–L5 posterior fusion, L2–3 laminectomy, and L3 corpectomy and cage. A left chest tube was also placed, and the patient was brought to the SICU. During her hospital course, she was found to have a small pulmonary embolism, and an IVC filter was placed by interventional radiology in preparation for orthopedic surgery, open reduction internal fixation of the right acetabular anterior column and ilium on hospital day 7. Since admission, the patient remained unable to move both lower extremities, though she did gradually regain sensation. The patient admitted to jumping intentionally, which was followed by a psychiatric evaluation during her hospital stay. She was evaluated by physical medicine and rehabilitation and was discharged to the spinal cord injury inpatient rehab on hospital day 33.

### 3.4. Case 4

#### 3.4.1. Presentation

A 33-year-old male with a past medical history of alcohol use disorder was brought in by EMS after reportedly being found near the LIRR tracks after having been struck by a train and flung off the tracks. Multiple facial lacerations were noted on arrival, with a bloody airway in the trauma bay. Initial GCS was 7, and the patient was intubated. Initial FAST was negative, but the patient then became hypotensive. A repeat FAST exam was positive in the suprapubic region, mass transfusion protocol was activated, and the patient was taken directly to the operating room with trauma surgery. On exploratory laparotomy, the patient was found to have multiple large splenic lacerations and a serosal tear of the transverse colon. The patient underwent splenectomy, repair of the transverse colon serosal tear, and left chest tube placement for a small left pneumothorax.

#### 3.4.2. Imaging

**Figure 4:**
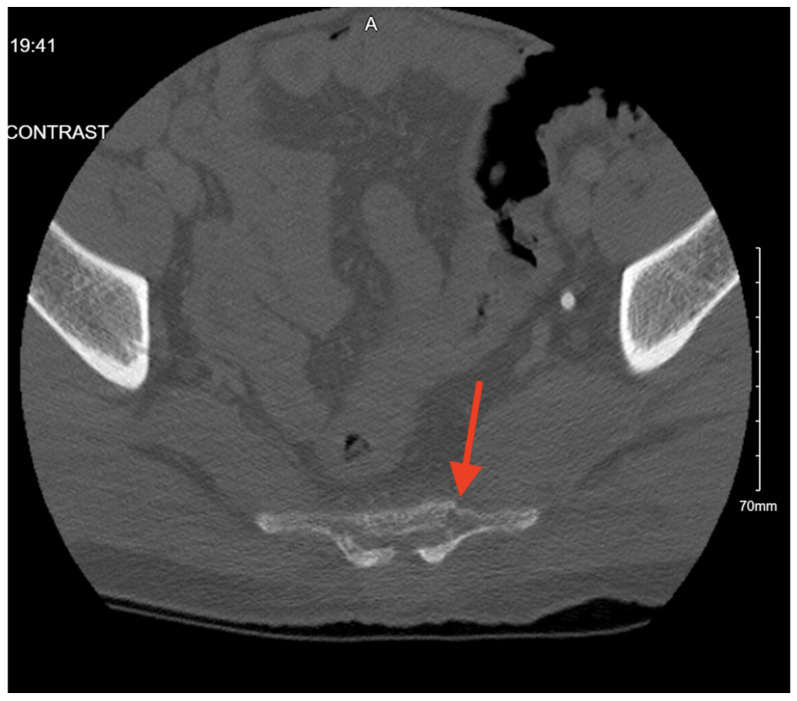
CT abdomen/pelvis images of the described fracture in case 4. Red arrows are used to indicate the location of pelvic fractures for easier identification.

Pelvic imaging performed after surgery showed a transverse S4 fracture (Figure 4). He also had multiple bilateral rib fractures, a left scapula comminuted displaced fracture, multiple cervical, thoracic, and lumbar spine fractures, and facial fractures.

#### 3.4.3. Management and Outcome

The patient was admitted to the surgical ICU for close monitoring and management of critical polytraumatic injuries. He had a worsening neurological exam; repeat CTH demonstrated small subdural hygromas and interval development of a small amount of layering subdural hemorrhage. Neurosurgery recommended non-surgical management for the pelvic and spinal fractures. His hospital course was complicated by bacterial fluid collection in the pelvis, requiring antibiotics. The patient was ultimately discharged to acute rehab on hospital day 30.

### 3.5. Case 5

#### 3.5.1. Presentation

A 95-year-old female with a past medical history of hypertension and chronic kidney disease was brought in by EMS with her daughter after a fall 3 days prior when going down the stairs, landing on her right side and striking her head. She went to an outside hospital that day and had imaging that showed no acute pathology. She was discharged home but had not been able to stand, sit, or ambulate since the incident. In the ER, the patient was complaining of right upper back pain, shoulder pain, and hip pain. On exam, she had a right forehead abrasion, and bruising was noted to the right shoulder, but she had no gross deformities. Her FAST was negative for any free fluid.

#### 3.5.2. Imaging

**Figure 5:**
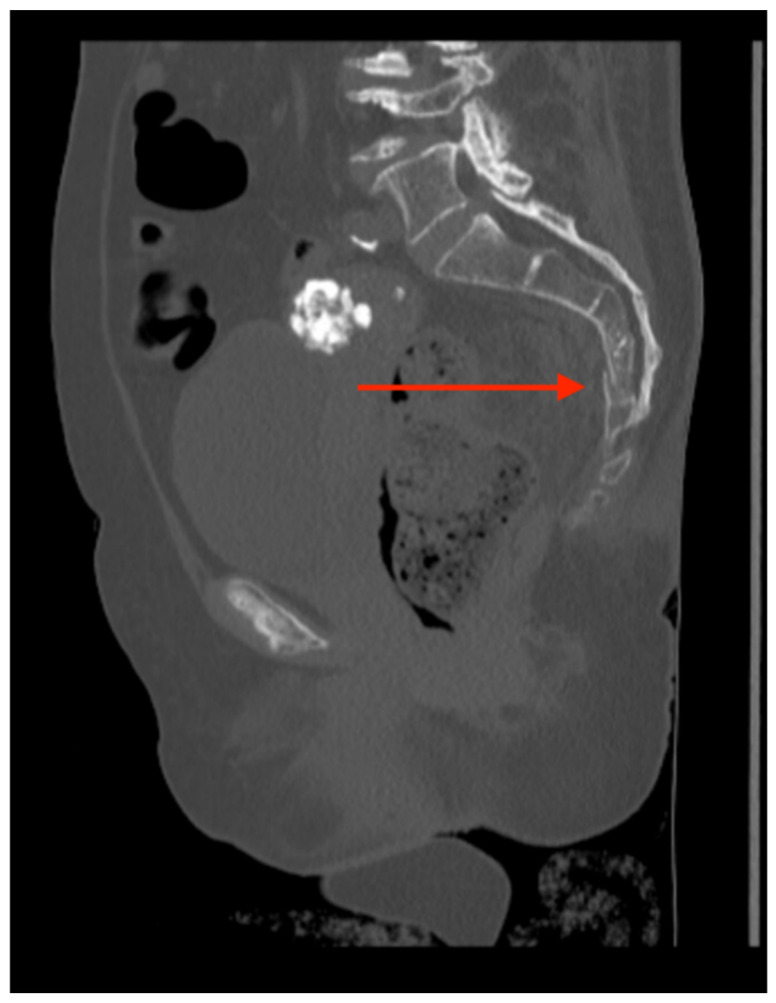
CT abdomen/pelvis images of the described fracture in case 5. Red arrows are used to indicate the location of pelvic fractures for easier identification.

Pelvic imaging revealed a nondisplaced sacral fracture, approximately at the level of S4 (Figure 5). She was also found to have a small acute subdural hemorrhage, a non-displaced fracture of the right distal clavicle, and fractures of the right 3rd and 4th ribs.

#### 3.5.3. Management and Outcome

Neurosurgery recommended no surgical interventions for her sacral fracture. Orthopedic surgery likewise recommended non-surgical management of her clavicle fracture. Physical medicine and rehabilitation was consulted and recommended that the patient start physical and occupational therapy. She was discharged to a subacute rehab on hospital day 14.

### 3.6. Case 6

#### 3.6.1. Presentation

An 86-year-old male with a past medical history of thyroid disease, hypertension, and coronary artery disease on apixaban presented to the emergency department after an unwitnessed fall. Per family, they heard a thud and attended to the patient, who was responsive and following commands. The family states he fell two steps with a head strike and loss of consciousness. On arrival, the patient had reported worsening in his mental status. On exam, GCS was 14, he had a laceration and hematoma to the posterior scalp, no hemotympanum, and dried blood in the nares. The pelvis had no visible injury and was stable to compression.

#### 3.6.2. Imaging

**Figure 6:**
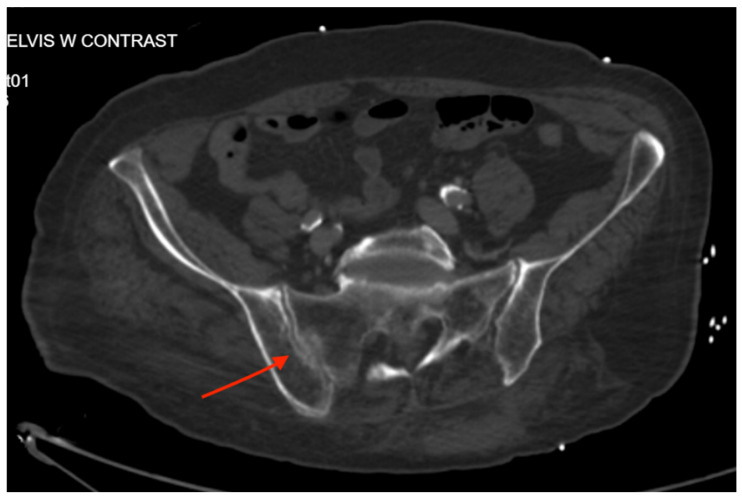
CT abdomen/pelvis images of the described fracture in case 6. Red arrows are used to indicate the location of pelvic fractures for easier identification.

Pelvic imaging showed acute nondisplaced bilateral sacral alar fractures with extension into the sacroiliac joint on the right and probable involvement of the right S3 neural foramen (Figure 6). He was also found to have a hyperacute left holohemispheric subdural hematoma with extensive rightward midline shift, left uncal herniation, and extensive scattered subarachnoid hemorrhage along the bifrontal regions and left frontotemporal region.

#### 3.6.3. Management and Outcome

In the ED, GCS declined to 11, the patient was then found to have a fixed and dilated left pupil and was immediately brought in for a CT scan due to concern for intracranial bleeding, and neurosurgery was consulted. While in the CT scanner, the patient had a decline in GCS to 8. The CT head non-contrast showed a traumatic brain injury, showing significant intracranial bleeding. His family was informed and chose to pursue medical management and forego surgical intervention and endotracheal intubation. The patient was admitted to the surgical ICU for monitoring. He was made DNR/DNI and continued comfort care until he was pronounced dead on hospital day 6.

### 3.7. Case 7

#### 3.7.1. Presentation

A 52-year-old male with an unknown past medical history was brought in by EMS after being found down at Rikers at the bottom of many steps, presumed to be a possible suicide attempt. When the paramedics arrived, they reported that the patient’s GCS was 3, and they had difficulty intubating the patient in the field. On arrival to the emergency department, the patient was breathing spontaneously but was unresponsive and unable to provide any history. On primary evaluation, the patient was unable to protect his airway, so he was intubated. His initial GCS was 3 with nonreactive pupils and was not withdrawing to noxious stimuli. On exposure, he had an obvious right lower extremity deformity and an abrasion and hematoma to the right side of the head. His pelvis was stable to compression and had no visible injuries. His FAST was negative.

#### 3.7.2. Imaging

**Figure 7:**
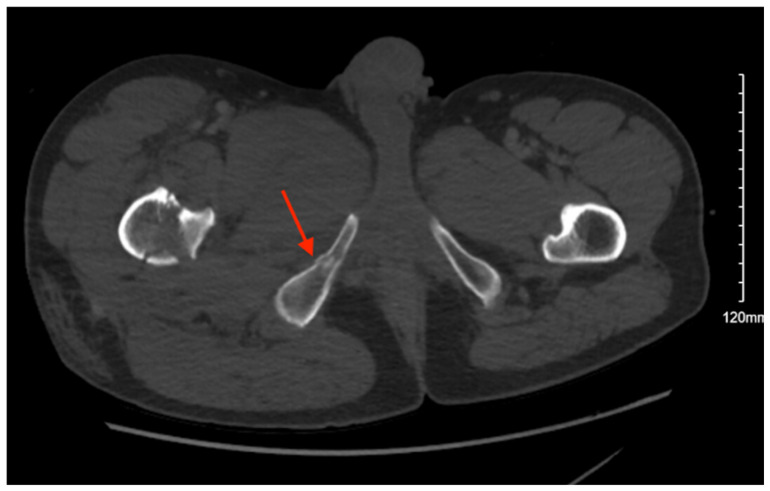
CT abdomen/pelvis images of the described fracture in case 7. Red arrows are used to indicate the location of pelvic fractures for easier identification.

Pelvic imaging showed a nondisplaced fracture of the right inferior pubic ramus (Figure 7). Additional injuries included subtrochanteric fracture of the right proximal femur, extensive diffuse subarachnoid hemorrhage, multiple small subdural hematomas, mild intraventricular hemorrhage, nondepressed fracture of the right frontal parietal calvarium, right internal carotid artery dissection with occlusion at the skull base, and fracture of the transverse processes of L2 and L3.

#### 3.7.3. Management and Outcome

Given his hypotension and injuries, the patient was started on a massive transfusion protocol. The orthopedic surgery team placed a pin to stabilize the right femur fracture in the ED. Neurosurgery diagnosed the patient with diffuse anoxic–hypoxic brain injury and irreversible ischemic changes and injury. They recommended no aggressive treatment beyond supportive care. Neurological determination of death was made on hospital day 3.

### 3.8. Case 8

#### 3.8.1. Presentation

An 85-year-old female with a past medical history of Parkinson’s, orthostatic hypotension, and right greater trochanteric fracture status post open reduction internal fixation presented to the ED after a fall from standing at home. The patient reportedly fell onto her right side, hitting her right shoulder, wrist, hip, ankle, and right side of her head. She denied any loss of consciousness. On evaluation, GCS was 15, she had a right lateral forehead laceration, stable pelvis, and her cervical, thoracic, and lumbar spines were non-tender, without any palpable step-offs.

#### 3.8.2. Imaging

**Figure 8:**
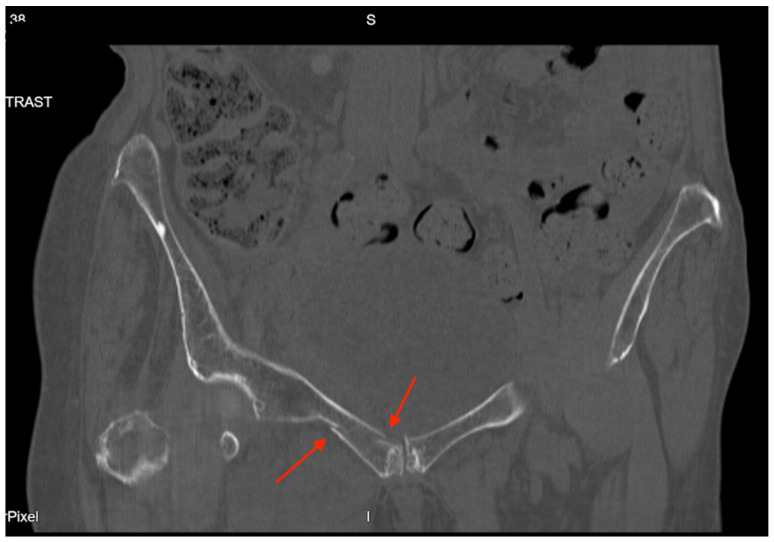
CT abdomen/pelvis images of the described fracture in case 8. Red arrows are used to indicate the location of pelvic fractures for easier identification.

Pelvic imaging showed an acute fracture of the superior and inferior pubic rami on the right with associated hematoma and possible fracture of the right sacral ala (Figure 8). Additional injuries included an acute subdural hemorrhage over the right cerebral hemisphere with mass effect on the right lateral ventricle, with a midline shift to the left of approximately 6 mm.

#### 3.8.3. Management and Outcome

Orthopedic surgery was consulted and recommended that the patient bear weight as tolerated on bilateral lower extremities and stated that there was no plan for any surgical interventions. Neurosurgery recommended seizure prophylaxis and repeat CT head; no acute neurosurgical interventions. The patient was admitted to the STICU for management of her traumatic injuries. Physical and occupational therapy were consulted, and it was recommended that when medically stable, the patient should be discharged to an acute rehab facility, which she was on hospital day 14.

### 3.9. Case 9

#### 3.9.1. Presentation

An 86-year-old female with a past medical history of hypertension and breast cancer status post left mastectomy presented to the ED via EMS after getting hit by a car and subsequently landing on her right side. She reported hitting her head and loss of consciousness. On arrival, the patient was complaining of lower back pain, bilateral hip pain, and dizziness. Examination showed a GCS of 15, and she followed commands. A hematoma was palpated at the left occiput. Her neck and chest had no visible injuries and no bony instability. Her pelvis was stable to compression and had no visible injuries. She had a visible right thigh contusion that appeared old. On examination of her back, she was tender to palpation of the midline lumbar and sacral spine. There were no step-offs or deformities noted. Her FAST was negative.

#### 3.9.2. Imaging

**Figure 9:**
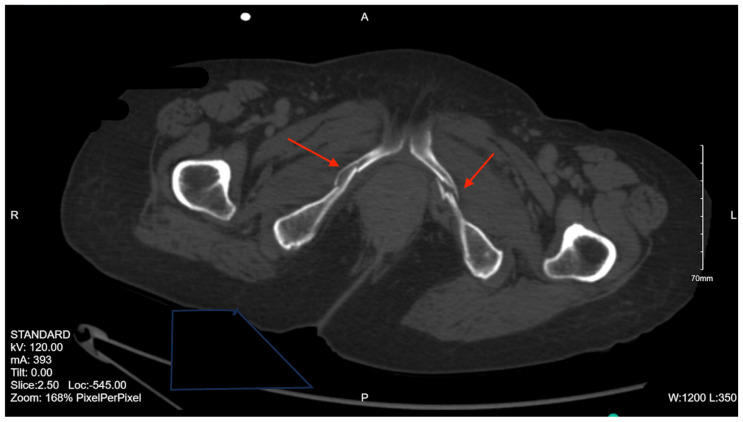
CT abdomen/pelvis images of the described fractures in case 9. Red arrows are used to indicate the location of pelvic fractures for easier identification.

Pelvic imaging showed an acute fracture of the left superior pubic ramus near the symphysis and the bilateral inferior pubic rami and an acute fracture of the anterior aspect involving the lateral mass of S1 (Figure 9). She was also found to have a subcapital fracture of the right hip and a fracture involving the anterior aspect of the vertebral body of L5.

#### 3.9.3. Management and Outcome

Repeat CT head showed an enlarging intraparenchymal bleed within the right temporal lobe with mass effect and an enlarging right parietal subdural bleed, and a small focus of subarachnoid blood within the medial portion of the right occipital lobe. The patient was subsequently admitted to the STICU for further management of her traumatic injuries. Orthopedic surgery recommended that the patient bear weight as tolerated and undergo non-surgical management for the pelvic fractures. Neurosurgery recommended seizure prophylaxis and continued CT monitoring. She was evaluated by a physical medicine and rehabilitation professional who recommended physical and occupational therapy. She was discharged to an acute rehab facility on hospital day 7.

### 3.10. Case 10

#### 3.10.1. Presentation

A 33-year-old male with a past medical history of schizophrenia and opioid use disorder was BIBEMS after a fall from a subway overpass. On exam, GCS was 13, and he was noted to have inappropriate affect. His left pupil was minimally reactive to light, but his extraocular movements were intact. His chest had an abrasion to the mid-chest but no visible deformity. A right knee effusion was noted. On examination of his back, he had multiple abrasions on his right flank and upper back, with associated lumbar spine midline tenderness to palpation. His FAST was negative.

#### 3.10.2. Imaging

**Figure 10:**
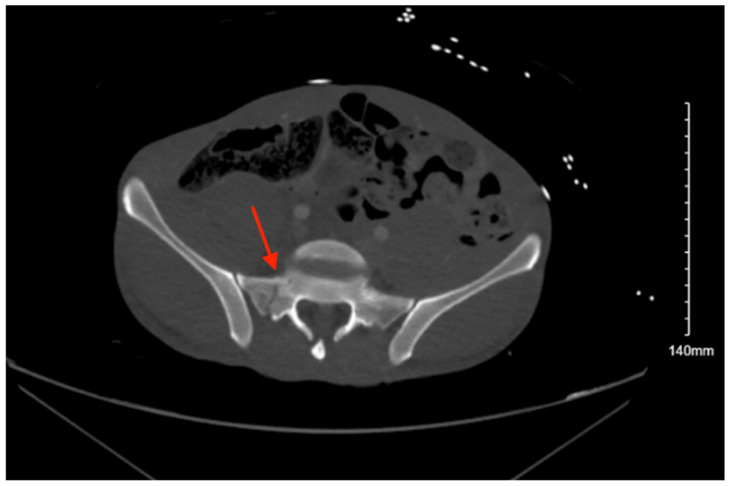
CT abdomen/pelvis images of the described fractures in case 10. Red arrows are used to indicate the location of pelvic fractures for easier identification.

Pelvic imaging showed an acute right sacral ala fracture, a nondisplaced acute right inferior pubic ramus fracture, and a nondisplaced acute fracture involving the lateral aspect of the right acetabulum (Figure 10). Additional imaging showed bilateral pneumothoraces, a small right hemothorax, fractures involving the posterior 9th through 12th ribs, grade II hepatic injury, concern for renal contusion, and L1–5 transverse process fractures. There was also retroperitoneal fluid surrounding the upper abdominal aorta without contrast blush and a linear flap within the upper abdominal aorta at the level of the celiac and SMA origins, concerning for traumatic injury.

#### 3.10.3. Management and Outcome

A right chest tube was placed in the ED. Given the finding of suspected aortic injury from the CT abdomen pelvis with contrast, a CT angiography of the abdomen pelvis was performed, which showed no evidence of vascular abnormality. The patient was admitted to the STICU for continued management of his traumatic injuries. Neurosurgery and orthopedic surgery recommended non-surgical management for the sacral and pelvic fractures. Psychiatry was consulted, given concern that his injury could have been a suicide attempt. The physical medicine and rehabilitation team was consulted and recommended that the patient start physical and occupational therapy. The patient was deemed medically optimized on hospital day 14 and was discharged with crutches to a shelter.

### 3.11. Case 11

#### 3.11.1. Presentation

A 37-year-old male with a past medical history of polysubstance use presented to the ED after a fall from over 25 feet. It was reported that the patient was running away from the police and tried to jump onto a pole to scale down from the 4th floor of a building, but missed and fell, with a reported loss of consciousness. On examination, he had a GCS of 15, left eyelid swelling and ecchymoses, and dried blood in his nares with no nasal septal hematomas. His chest and back had no obvious deformities and were non-tender to palpation. His abdomen was soft and non-distended but had some suprapubic tenderness to palpation. He had ecchymoses to the left elbow and had significant swelling and tenderness to palpation. His left lower extremity was shortened and externally rotated. A left knee abrasion and tenderness to palpation at the left hip and foot were present. His FAST was negative. A portable pelvis X-ray showed multiple fractures, so a pelvis binder was placed.

#### 3.11.2. Imaging

**Figure 11:**
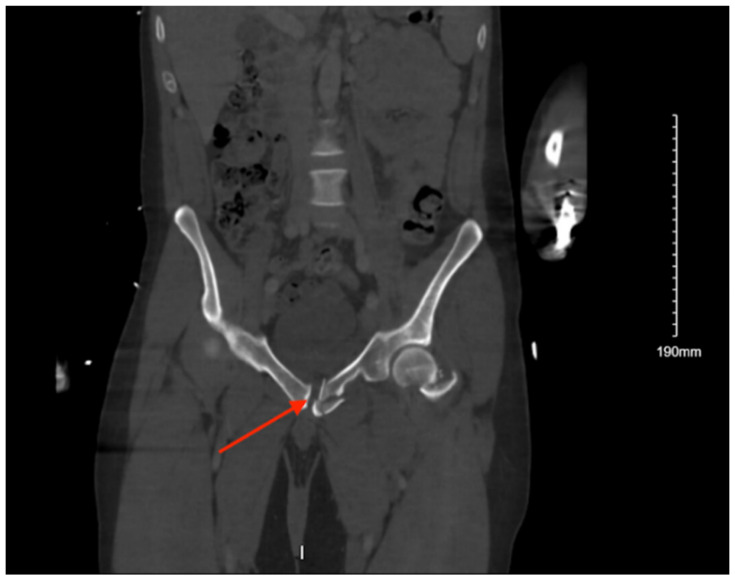
CT abdomen/pelvis images of the described fractures in case 11. Red arrows are used to indicate the location of pelvic fractures for easier identification.

Further pelvic imaging showed a left sacral nondisplaced fracture extending through the neural foramina and mild widening of the SI joint, incomplete hairline fracture of the iliac bone medially just above the acetabulum, left pubis, most medial superior ramus, and more extensive left inferior pubic ramus fractures, and anterior perivesical and left pelvic hematomas (Figure 11). He was also found to have a left frontal bone fracture, orbital and nasal bone fractures, a comminuted fracture of the left femoral neck, a proximal ulnar olecranon acute comminuted fracture, and an acute fracture of the radial neck.

#### 3.11.3. Management and Outcome

The patient was admitted to the STICU. Neurosurgery and orthopedic surgery recommended non-surgical management for the pelvic and sacral fractures. He underwent closed reduction internal fixation with pinning of the left femoral neck fracture on hospital day 2 with orthopedic surgery, obliteration of the frontal sinus and closed reduction of nasal bone on hospital day 5 with oral surgery, and open reduction internal fixation of the left olecranon on hospital day 8 with orthopedic surgery. He was followed by physical and occupational therapy during his stay, and they recommended that the patient be discharged to a sub-acute rehab facility, which he was on hospital day 41.

### 3.12. Case 12

#### 3.12.1. Presentation

An 86-year-old female with a past medical history of dementia was brought in by family after an unwitnessed fall; the patient was found at the bottom of the stairs by her husband. It was unclear how many steps she had fallen, and she was unable to provide further history, given her severe dementia. On exam, her GCS was 14, and per her family, she was at her neurologic baseline. Her chest had no visible injuries but was tender to palpation on the right side without crepitus. She had tenderness to palpation to the left shoulder, elbow, and wrist without any obvious deformities. She also had tenderness to palpation at the right hip, but her right lower extremity was not externally rotated or shortened. Her FAST was negative.

#### 3.12.2. Imaging

**Figure 12:**
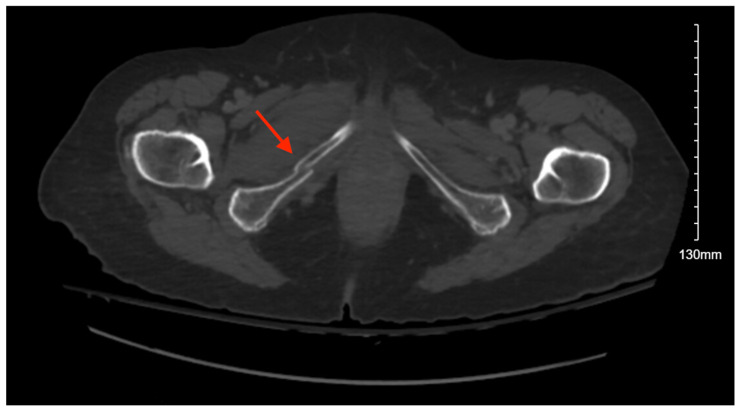
CT abdomen/pelvis images of the described fractures in case 12. Red arrows are used to indicate the location of pelvic fractures for easier identification.

Pelvic imaging showed right superior and inferior rami fractures and adjacent pelvic muscular enlargement likely secondary to intramuscular hematoma, right pubic symphysis fracture with adjacent hematoma, and a nondisplaced right sacral ala fracture (Figure 12). She was also found to have small quantities of scattered subarachnoid hemorrhage over the cerebral convexities, small acute subdural hematoma in the interhemispheric fissure without significant mass effect, nondisplaced right T8–10 transverse process fractures, multiple displaced and nondisplaced right rib fractures, and a left radial head fracture.

#### 3.12.3. Management and Outcome

Repeat CT head was stable with some improvement in the acute subdural hematoma. Orthopedic surgery recommended nonoperative management for the pelvic fractures. The physical medicine and rehabilitation team was consulted, and they recommended starting physical and occupational therapy. She was discharged to subacute rehab on hospital day 15.

### 3.13. Case 13

#### 3.13.1. Presentation

A 28-year-old female with no significant past medical history was brought in by EMS after being struck by a vehicle going at 15–45 miles per hour. Per the report, she flew 15 feet and lost consciousness. On arrival, her GCS was 6, and while in the ED, she was intubated and bilateral chest tubes were placed for tension hemopneumothoraces. The patient rapidly declined and became hypotensive to 80s/40s, and was initiated on mass transfusion protocol. On further examination, she had epistaxis and blood in the oropharynx. Her right pupil was fixed and dilated. Her pelvis was stable to compression. On examination of her extremities, she was withdrawing from pain in her upper and lower extremities, with distal pulses palpable and intact.

#### 3.13.2. Imaging

**Figure 13:**
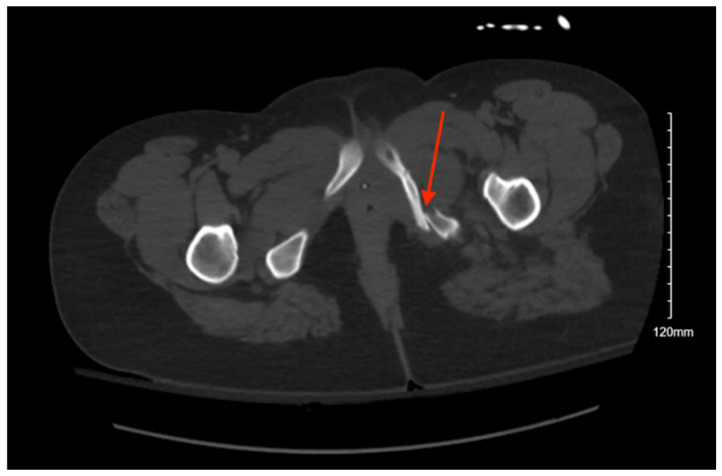
CT abdomen/pelvis images of the described fractures in case 13. Red arrows are used to indicate the location of pelvic fractures for easier identification.

Pelvic imaging showed acute avulsed fracture of the superior-lateral acetabulum, multi-comminuted fractures with distracted bony fragments left in the acetabulum, and an acute fracture of the medial aspect of the left superior pubic ramus (Figure 13). Other injuries included a non-depressed right frontotemporal skull fracture, right mastoid fracture, right clavicular fracture, and multiple bilateral rib fractures.

#### 3.13.3. Management and Outcome

Given the fractures on CT and her decompensation, interventional radiology was consulted, and the patient was taken to the IR suite for a pelvic angiogram and embolization. During the angiogram, they found no evidence of extravasation or pseudoaneurysm and successfully prophylactically embolized the left hypogastric, given the mechanism of injury. She was then transported to the STICU for management of her traumatic injuries. Orthopedic surgery was planned for an open reduction internal fixation of the left acetabulum once the patient stabilized. Her hospital course was complicated by worsening subarachnoid hemorrhage, causing elevated intracranial pressure requiring an external ventricular drain. She also developed a new pneumothorax requiring chest tube placement and pneumonia requiring antibiotics. She had a tracheostomy on hospital day 10. She later developed a left frontal hemorrhage with mass effect and underwent a left decompressive craniectomy with evacuation of left frontal intracranial hemorrhage and placement of a left external ventricular drain. Per the orthopedic surgery team, the patient was determined to be out of the surgical repair window on hospital day 39 due to her continued tenuous neurosurgical issues, so they recommended nonoperative management. She had minimal neurologic improvement, and on hospital day 58, she was deemed stable for discharge to a long-term acute care hospital. Summary of all cases presented can be found in Table 1 of this case series.

## 4. Management and Outcome

Of the 13 cases presented, six patients were male and seven were female. The ages ranged from 18–95 years old. The mechanisms of injury included falls and pedestrians struck by motor vehicles/trains. Of the fall patients, five had a fall from height, and four fell from standing. Of the patients who fell from standing, all were over the age of 85. All patients had injuries in addition to the pelvic fractures, many of which required management. A total of 10 patients had TBIs, 10 had fractures other than in the pelvis or sacrum, 4 required mass transfusions, and 5 required surgeries not related to the pelvic or sacral fractures, including open reduction internal fixation of the upper and lower extremities, an exploratory laparotomy with splenectomy and colon repair, and a craniectomy. One patient had visceral injuries that did not require surgical treatment, two underwent tracheostomy and PEG, and four required chest tubes.

In the immediate management of pelvic injuries, cases 3 and 11 had pelvic binders placed. Cases 2 and 13 underwent interventional radiology angiograms, and though neither showed evidence of acute bleeding, case 13 did have prophylactic embolization of the hypogastric artery. As far as surgical management, case 1 underwent percutaneous left SI screw placement and case 3 underwent open reduction internal fixation of the right acetabular anterior column and ilium. All of the remaining 11 cases were managed non-surgically. Of these, cases 2, 6, 7, and 13 could have possibly benefited from surgical repair, but it was not recommended due to the severity of their other injuries. In terms of disposition, all of the cases except case 10 required further inpatient medical treatment after discharge. Two patients went to acute rehab, five patients went to subacute rehab, three patients went to specialized programs (case 2 went to TBI rehab, case 3 went to SCI rehab, and case 13 went to long-term inpatient rehab), and two patients were declared dead.

## 5. Discussion

Previous literature shows that the majority of pelvic fractures are the result of high-energy impacts, most commonly motor vehicle collisions and pedestrians struck by vehicles [17,18,19,20]. Another common mechanism is a fall from height [1,9,14,15]. This is represented in the cases presented, with nine of the patients having a high impact mechanism (five fall from height, three struck by a vehicle, one struck by a train). Interestingly, none of the cases presented had injuries as a result of being an occupant in a motor vehicle crash, likely due to transport modalities utilized in New York City, where these cases occurred. The remaining four cases were the result of lower-energy impacts, either falls from standing or falls a few steps. All of the patients who sustained these low-energy injuries were over the age of 85. This is consistent with previous findings of increased incidence of pelvic fractures caused by falls from standing in elderly populations [21,22,23,24].

All cases had injuries in addition to pelvic fractures, including injuries to the head, chest, abdomen, spine, and extremities. It is well known that due to the high-energy mechanism of injury in many pelvic fractures, they are often associated with injuries to other areas of the body [1,12,15,25,26]. The severity of these additional injuries often impacts the course of treatment of pelvic injuries. For example, in case 13, the initial recommendation by orthopedic surgery was for the patient to undergo open reduction internal fixation, but due to the severity of the patient’s other injuries, this was deferred until the patient was outside of the window to have surgery. Head injuries specifically are common in polytrauma patients [14], which was shown in the cases presented, with 10 having TBIs. There are no definitive guidelines for treating pelvic fractures with concomitant head injuries, and the literature presents conflicting evidence. Some suggest that early fracture fixation might be harmful to patients with brain injuries, but others do not agree with these claims, suggesting that certain patients who did not have early fracture stabilization have worse outcomes [27,28,29,30]. One of the major concerns is the potential for blood pressure fluctuations during orthopedic surgery, causing additional brain injury [31]. This further highlights the importance of having a multidisciplinary team to care for these patients.

Management of patients with pelvic fractures largely depends on hemodynamic stability. At initial presentation to the hospital, it is widely accepted that hemodynamically unstable patients with suspected pelvic injury should have a pelvic binder placed [6,14,32,33]. Pelvic binders can also be placed in hemodynamically stable patients with unstable pelvic fractures as a bridge to mechanical fracture stabilization [14]. After pelvic binder placement, it is recommended that unstable patients get a chest and pelvic X-ray as well as FAST workup to identify potentially life-threatening injuries and identify possible sources of bleeding [3,6,14,34,35,36]. In this case series, all patients except one underwent pelvic X-ray as part of their initial workup in the ED; case 4 did not due to their hemodynamic instability and positive FAST necessitating immediate OR intervention. Of the 10 patients who had a FAST exam, only case 4 was positive, with free fluid in the suprapubic region. Some authors caution the use of the FAST exam in trauma patients with pelvic fractures, finding significantly reduced sensitivity and specificity [9,37,38,39,40,41]. Montmany Vioque et al. [9] found a false positive rate of 30.9% with over half undergoing unnecessary laparotomy, and a false negative rate of 34.8%, resulting in a delay of treatment. While this should by no means eliminate the use of FAST, clinicians should be aware of the reduced sensitivity and specificity in patients with pelvic fractures. When stable, all patients should receive a CT scan, ideally with contrast, to inventory all injuries and identify sources of bleeding [3,6,14,34,35,36].

All patients in this case series underwent CT abdomen–pelvis to determine the extent of their pelvic injuries in the ED, except case 4, who underwent CT after being stabilized post-exploratory laparotomy. This case series highlights the importance of obtaining a CT abdomen pelvis to recognize the full extent of the injuries present; six patients had initial pelvic X-rays that showed no evidence of fractures that were later identified on CT. This follows recent literature which has shown that the sensitivity of X-rays in detecting pelvic fractures ranges from 64.4–88.4% [42,43,44] when compared to CT, though it can be as low as 10.7% in detecting certain pelvic fractures (in this case ischial) [42]. These findings suggest diminishing utility in pelvic X-rays as a diagnostic tool in the age of CT, especially for sacral and iliac fractures [43,45]. Rather, pelvic X-rays can be beneficial as part of a management decision algorithm in unstable patients to identify those who may benefit from interventions like a pelvic binder, but in stable patients it is unlikely to provide much utility, especially for patients that will undergo CT imaging [42,46].

Further initial management of pelvic injuries can include angioembolization, external fixation, and preperitoneal pelvic packing. Two patients in the case series underwent angiography with IR for possible embolization, and though neither showed signs of active arterial bleeding, case 13 did have prophylactic embolization of the hypogastric artery. Angioembolization is widely recognized as the main treatment for acute bleeding control [47,48,49,50,51,52,53,54,55,56,57], though it is important to note that embolization is largely only effective for arterial injury and does not control bleeding from venous or bony sources [6].

Definitive management of pelvic fractures can be non-operative or surgical stabilization. Case 1 underwent percutaneous left SI screw placement on hospital day 9 and case 3 underwent open reduction internal fixation of the right acetabular anterior column and ilium on hospital day 7. The timing of internal pelvic fixation often depends on the patient’s other injuries and overall stability. For stable patients, early fixation has shown improved outcomes and reduced risk of complications [58,59,60]. For more severely injured patients who required prolonged resuscitation efforts, delaying surgery for at least four days can significantly decrease complication rates [61,62,63]. The remaining 11 cases were managed non-operatively, though four of these cases (2, 6, 7, and 13) might have been candidates for surgical repair if not for the severity of their other injuries. Case 13 had originally planned for open reduction internal fixation of the left acetabulum but was determined to be outside of the surgical repair window. Protocols for what this “surgical window” actually is vary by individual location, though guidelines suggest that repair within 21 days is associated with better long-term results [64]. The primary goal in pelvic fractures is functional rehabilitation and weight bearing as soon as possible to avoid complications associated with prolonged immobilization [14,65]. A multidisciplinary team, including trauma surgeons, neurosurgeons, orthopedic surgeons, and physical/occupational therapists, can be extremely beneficial in determining the best course of action to accomplish this goal.

One new approach to definitive treatment of pelvic fractures that is becoming increasingly prevalent is the use of 3D printing for preoperative planning. With the complexity of some pelvic fractures, preoperative assessment can be difficult, even with the advent of 3D reconstructive CT imaging [66]. 3D printing can improve visualization, and assist in surgical approach planning and even pre-contouring hardware to fit individual patient needs [67]. Solyom et al. [66] found that 3D model printing shortens the duration of surgery, reduces intraoperative complications, and improves clinical outcomes. They did also note, though, that model printing can take 22 h, which may limit its functionality in emergent cases.

## 6. Strengths and Limitations

This case series offers several strengths, including comprehensive data collection that provides detailed insights into patient demographics, mechanisms of injury, and management strategies for pelvic fractures and associated injuries. The study underscores the importance of a multidisciplinary approach, by incorporating perspectives from trauma surgeons, neurosurgeons, orthopedic surgeons, and rehabilitation specialists. This reflects real-world collaboration in complex trauma care and supports the growing emphasis on team-based management in improving patient outcomes. Additionally, the discussion of various diagnostic and treatment modalities, such as pelvic binders, angiography, and both surgical and non-surgical interventions, offers practical insights into clinical decision-making scenarios, particularly in patients with competing priorities such as traumatic brain injuries or hemodynamic instability.

However, this study has limitations that must be acknowledged. The small sample size of only 13 cases restricts the generalizability of the findings to broader populations. As a single-center, retrospective case series, this study is subject to selection bias and limitations in data completeness, particularly regarding long-term follow-up and functional outcomes. The retrospective design also restricts control over confounding variables and precludes definitive causal conclusions about the effectiveness of specific interventions. Furthermore, the relatively low number of patients undergoing surgical fixation reduces the ability to draw meaningful comparisons between operative and non-operative management strategies. Finally, variability in documentation and clinical decision-making may reflect institutional practices and limit the broader applicability of certain findings.

## 7. Conclusions

This case series underscores the complexity of managing pelvic fractures, particularly in polytrauma patients. The findings reaffirm the association between high-energy mechanisms and severe concomitant injuries while highlighting the prevalence of low-energy fractures in the elderly. The variability in management approaches, including the use of pelvic binders, angiography, and surgical stabilization, demonstrates the importance of case-specific care. This study emphasizes the role of a multidisciplinary team in optimizing outcomes, particularly in patients with competing priorities, such as traumatic brain injuries or hemodynamic instability. Future studies should aim to definitively determine the utility of pelvic X-rays in both stable and unstable patients, explore further uses of 3D printing for complex pelvic injuries, and develop standardized care pathways that integrate multiple services, including trauma and orthopedic surgery, interventional radiology, and rehabilitation services to improve coordination and outcomes.

## Figures and Tables

**Table 1:** Summary of cases presented.

Case	Age/Sex	Injury Mechanism	Fractures	Intervention	Additional Injuries
1	52M	Fall > 25 feet	Right superior pubic ramus, left inferior pubic ramus, left sacral wing	Percutaneous left SI screw with L5 instrumentation	Multiple facial fractures, left radius fracture, left femur fracture, left tibial shaft fracture
2	62F	Pedestrian struck by vehicle	Impacted left superior and inferior pubic rami, bilateral sacral ala	Non-operative	Subarachnoid hemorrhage, right tibial plateau fracture
3	18F	Fall > 30 feet	Displaced distal sacrum, comminuted right iliac, displaced right inferior pubic ramus, displaced right acetabulum	Open reduction internal fixation of the right acetabular anterior column and ilium	Subdural hematoma, compression fractures of T6–8, T11, and T12, L2, and L3 burst fractures, 4th–6th left rib fractures, left pneumothorax
4	33M	Pedestrian struck by train	Transverse S4	Non-operative	Splenic lacerations, transverse colon serosal tear, pneumothorax, subdural hygroma, and hemorrhage
5	95F	Fall from standing	Nondisplaced sacral fracture	Non-operative	Subdural hemorrhage, right distal clavicle fracture, right 3rd and 4th rib fractures
6	86M	Fall from standing	Bilateral sacral ala with extension into the right sacroiliac joint	Non-operative (family decision)	Subdural hematoma, subarachnoid hemorrhage, uncal herniation
7	52M	Fall down multiple stairs	Right inferior pubic ramus	Non-operative (irreversible brain injury)	Femur fracture, subarachnoid hemorrhage, subdural hematoma, intraventricular hemorrhage, right parietal fracture, right ICA dissection, L2 and L3 transverse process fractures
8	85F	Fall from standing	Superior and inferior pubic rami, right sacral ala	Non-operative	Subdural hemorrhage
9	86F	Pedestrian struck by vehicle	Left superior pubic ramus, bilateral inferior pubic rami, anterior S1	Non-operative	Right subcapital femur fracture, L5 vertebral body fracture
10	33M	Fall from subway overpass	Right sacral ala, right inferior pubic ramus, lateral right acetabulum	Non-operative	Bilateral pneumothoraces, right hemothorax, right 9th–12th rib fractures, grade II hepatic injury, renal contusion, L1–5 transverse process fractures
11	37M	Fall > 25 feet	Left sacral neural foramina, left superior and inferior pubic ramus, left pubis	Non-operative	Sacroiliac joint widening, frontal bone fracture, orbital and nasal fractures, left femoral neck fracture, olecranon fracture, radial fracture
12	86F	Fall down unknown stairs	Right superior and inferior rami, right pubic symphysis, right sacral ala	Non-operative	Subarachnoid hemorrhage, subdural hematoma, T8–10 transverse proves fractures, multiple right rib fractures, left radial fracture
13	28F	Pedestrian stuck by vehicle	Left multi-comminuted acetabulum, left superior ramus	Left hypogastric artery embolization, plan for open reduction internal fixation of left acetabulum deferred due to severity of other injuries	Bilateral hemopneumothoraces, right frontotemporal fracture, right mastoid fracture, right clavicular fracture, multiple bilateral rib fractures, subarachnoid hemorrhage

## Data Availability

The original contributions presented in this study are included in the article. Further inquiries can be directed to the corresponding author.
